# Extraordinary optical transmittance generation on Si_3_N_4_ membranes†

**DOI:** 10.1039/d3nr02834h

**Published:** 2023-09-29

**Authors:** Salvatore Macis, Maria Chiara Paolozzi, Annalisa D'Arco, Federica Piccirilli, Veronica Stopponi, Marco Rossi, Fabio Moia, Andrea Toma, Stefano Lupi

**Affiliations:** a Department of Physics, Sapienza University Piazzale Aldo Moro 5 00185 Rome Italy salvatore.macis@uniroma1.it; b INFN – Laboratori Nazionali di Frascati via Enrico Fermi 54 00044 Frascati Rome Italy; c Elettra – Sincrotrone Trieste S.C.p.A. S.S. 14 km-163 5 in Area Science Park I-34149 Basovizza Trieste Italy; d IOM-CNR, Area Science Park Strada Statale 14 km 163 5 34149 Basovizza TS Italy; e SBAI, Department of Basic and Applied Sciences for Engineering, University of Rome “La Sapienza” Via Scarpa 16 00161 Rome Italy; f Istituto Italiano di Tecnologia via Morego 30 Genova 16163 Italy

## Abstract

Metamaterials are attracting increasing attention due to their ability to support novel and engineerable electromagnetic functionalities. In this paper, we investigate one of these functionalities, *i.e.* the extraordinary optical transmittance (EOT) effect based on silicon nitride (Si_3_N_4_) membranes patterned with a periodic lattice of micrometric holes. Here, the coupling between the incoming electromagnetic wave and a Si_3_N_4_ optical phonon located around 900 cm^−1^ triggers an increase of the transmitted infrared intensity in an otherwise opaque spectral region. Different hole sizes are investigated suggesting that the mediating mechanism responsible for this phenomenon is the excitation of a phonon-polariton mode. The electric field distribution around the holes is further investigated by numerical simulations and nano-IR measurements based on a Scattering-Scanning Near Field Microscope (s-SNOM) technique, confirming the phonon-polariton origin of the EOT effect. Being membrane technologies at the core of a broad range of applications, the confinement of IR radiation at the membrane surface provides this technology platform with a novel light–matter interaction functionality.

## Abstract 2

Optical properties of Si_3_N_4_ membranes are modified *via* patterning of holes to excite surface phonon polaritons and increase transmittance.

## Graphical abstract

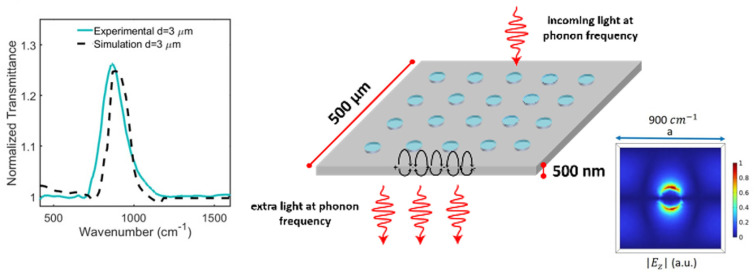

## 1. Introduction

Metamaterials are a class of artificially engineered systems exhibiting exotic electromagnetic and transport properties not available in natural materials.^1–3^ Interestingly, the electromagnetic and transport response of these media to external stimuli can be engineered at will, going beyond some of the limitations encountered when using natural materials. For these reasons, metamaterials are of particular importance in optics and photonics.^4–8^ Although less developed compared to plasmonic (electron-based) metamaterials, phononic metamaterials have been recently attracting increasing attention allowing, for instance, the control of the phonon propagation and heat transport at a microscopic level.^9–12^ Moreover, they are employed in several applications, working as mechanical filters,^13^ beam steerers, and lenses,^14^ reaching a similar level of feasible applications as plasmonic metamaterials do. One of the striking advantages concerning artificial materials relies on the possibility to manipulate electromagnetic radiation at a sub-wavelength scale, looking for field concentration and enhanced optical transmission.^15,16^ Indeed, highly localized fields in the vicinity of films can be beneficial for performing sensing studies, intensively investigated in nanophotonics.^17,18^ Although plasmonics has been devoting great attention to this kind of research, drawbacks are not missing. Indeed, in metallic nanostructures, the capability to concentrate light at sub-diffraction scales is efficient at near-infrared and visible frequencies, but in the infrared and terahertz (THz) range high attenuation due to inherent material absorption is experienced.^19–21^ Even noble metals, such as silver and gold, are scarcely efficient in this spectral range, which strongly affects the realization of such applications. Although the use of unconventional materials like graphene,^22–25^ topological insulators,^26^ and high temperature superconductors^27,28^ may mitigate this effect, an alternative way to obtain efficient field confinement and enhancement at IR frequencies consists in exploiting another kind of collective excitation: the surface phonon polariton (SPhP).^29^ Using polar dielectric materials, a coupling between electromagnetic radiation and optical phonons at infrared frequencies can be obtained in a way similar to both propagating and localized surface plasmons in metals.^30^ Advantageously, polar dielectrics allow to obtain simultaneously sub-diffraction confinement *via* a negative dielectric permittivity, and low optical losses in the THz–IR spectral range due to the small damping rate of optical phonons.^31^

In this work, we studied the Extraordinary Optical Transmittance phenomenon^32–35^ generated on phononic metamaterials obtained from a set of patterned self-standing silicon nitride (Si_3_N_4_) membranes. Membrane technologies show a broad range of applications embracing both industrial and fundamental research.^36,37^ Within this context, Si_3_N_4_ membranes offer an ultra-flat and mechanically robust support to Transmission Electron Microscopy (TEM) and Scanning Transmission Electron Microscopy (STEM) imaging, real-time chemical reactions, and crystal growth investigations. The development of the so-called *in situ*/*operando* spectroscopies has indeed led to major advances in functional material characterization, leveraging also on membrane-based technologies. In line with these characteristics, the possibility to confine and enhance electromagnetic (EM) waves at the membrane surface could inherently endow this technological platform with newly designed functionalities, where a stronger light–matter interaction mediated by a phonon-polariton field can play a crucial role.

In this framework, we fabricated periodic arrays of holes with different diameters *d* (3, 5, and 7 μm) with a lattice parameter *a* of 12 μm (see below) on Si_3_N_4_ membranes, and investigated for the first time, at least to our knowledge, their phonon-polariton properties in the IR spectral region. The fabricated metamaterial structure allows the coupling between the incoming infrared electromagnetic wave and a Si_3_N_4_ optical phonon around 900 cm^−1^, generating a surface phonon polariton at the air–Si_3_N_4_ interface. The scattering of this SPhP and its radiative decay through the hole array explain the appearance of an EOT peak in the same spectral region. The electric field distribution around the holes has been further investigated by numerical simulations and nano-IR measurements based on a Scattering-Scanning Near Field Microscope (NEASPEC s-SNOM). Both simulations and measurements confirm the phonon-polariton origin of the EOT effect.

## 2. Experimental

### 2.1. Si_3_N_4_ membranes synthesis and patterning

As already mentioned above, for this study we used self-standing silicon nitride membranes, which are widely used in applications such as TEM and STEM.^36,37^ Their main advantage consists in being chemically inert and mechanically robust, thus being good candidates to make a free-standing thin metamaterial structure.

The fabrication of these suspended Si_3_N_4_ membranes, provided by Istituto Italiano di Tecnologia (IIT) in Genova, can be summarized in the following steps. 500 μm thick double-side polished Si(100) wafer with 500 nm low stress Low Pressure Chemical Vapor Deposition (LPCVD) Si_3_N_4_ film, grown on both sides, was used. An etching window was realized on the wafer backside using standard optical lithography (Süss MicroTec MA6/BA6 mask aligner system). Before UV exposure, a S1813 positive resist was spun at 4000 rpm and baked for 1 minute at 95 °C. The exposed resist was developed in MICROPOSIT MF-319 for 45 s. The sample was then loaded into a plasma-enhanced reactive ion etching system (Sentech SI500), and processed with a 12 : 1 gas mixture CHF_3 _: O_2_, 600 W of ICP power and 30 W of RF power. The suspended membranes were finally realized using wet-etching in KOH. Focused ion beam (FIB) machining was employed to mill the Si_3_N_4_ surface with a nanometric spatial resolution. The process was performed in a FEI – Dual Beam Helios Nanolab 650 machine, where Ga^+^ ions were emitted with a current of 21 nA and accelerated by a voltage of 30 kV. Prior to ion milling, a 20 nm-thick Au film was evaporated by physical vapour deposition technique, in order to avoid charging effects. In this way, three patterned samples with a lattice parameter of 12 μm and 3, 5, 7 μm diameters were realized, covering a 300 × 300 μm^2^ area. The process was finalized by dissolving the Au sacrificial layer in a standard etchant potassium iodide solution.

### 2.2. Optical measurements

Optical transmittance measurements have been performed in a spectral region including terahertz (THz), IR, Visible, and Ultraviolet (UV), by combining different light sources, detectors, and optical elements. A Bruker Vertex 70v Fourier-Transform interferometer and a Jasco V-770 Spectrophotometer have been used to cover the wide THz-UV spectral range (150–52 000 cm^−1^) for the characterization of the unpatterned membrane. A resolution of 4 cm^−1^ was used on the THz–IR region and of 2 nm for the Visible-UV one. The Bruker interferometer coupled to a Hyperion-2000 infrared microscope (Cassegrain 15× objective with an illumination angle of 30° and a numerical aperture NA = 0.4) has been used to cover the THz and IR spectral range for the characterization of all the patterned samples. The use of a microscope was necessary due to the small size of the patterned area.

### 2.3. Simulations

Numerical simulations of the optical properties of the metamaterial array were performed through COMSOL Multiphysics *via* a finite-element method. The transmittance of the unpatterned and patterned samples was simulated in the spectral range between 400 and 1650 cm^−1^, allowing the extraction of the electric field distribution maps in and out of resonance conditions. The Si_3_N_4_ metamaterial array, surrounded by air, was simulated by applying periodic boundary conditions to a unit cell of period *a* = 12 μm and hole-diameter *d* = 3 μm, thus generating an infinite periodicity. The complex dielectric function and optical conductivity of the unpatterned sample have been used as input parameters.

### 2.4. Scattering-type scanning near-field optical microscopy measurements

In order to perform nano-resolved IR microscopy and AFM measurements, metallic tips (ARROW-NcPt from Nano World, Matterhorn, Switzerland) with a diameter of about 20 nm, nominal resonance frequency of 285 kHz, and force constant of 42 N m^−1^, were used. Measurements were performed in tapping mode at a tapping frequency Ω of 243.5 kHz. An infrared QCL tunable laser operating in the spectral region between 850 cm^−1^ and 1800 cm^−1^ was used. A nitrogen-cooled Mercury Cadmium Telluride (MCT) detector was used to detect the infrared signal. Phase and amplitude of the IR signal have been acquired directly from Neaspec acquisition software, Neascan, by lock-in demodulation of the interference signal at the 3rd-harmonic of the cantilever oscillation frequency, and by using as a reference a Si substrate. Data postprocessing was performed with Neaplotter (Neaspec), and a phase correction filter was applied in order to take into account the slight phase changes of the IR laser.

## 3. Results and discussion

**Fig. 1:**
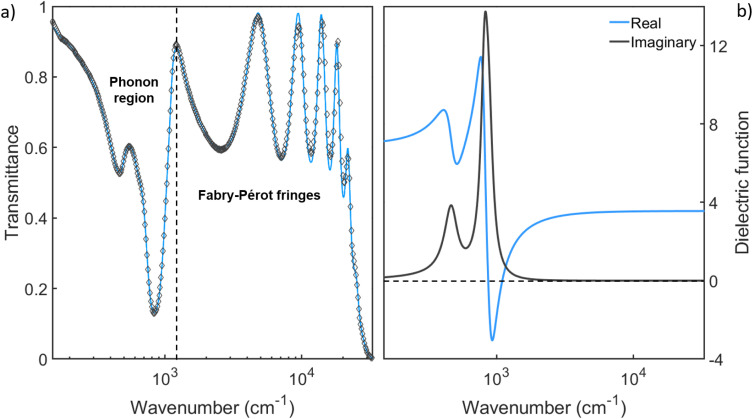
(a) Experimental (light blu line) and fitted (black empty diamond symbols) transmittance of the Si_3_N_4_ membrane, measured in the THz-UV range. The vertical black dashed line separates the phonon absorption region (low-frequency) to the high-frequency part characterized by Fabry–Pérot fringes. (b) Real (light blue line) and imaginary (black line) parts of the dielectric function, extracted from the fitted transmittance by considering the Fabry–Pérot effect.

**Fig. 2:**
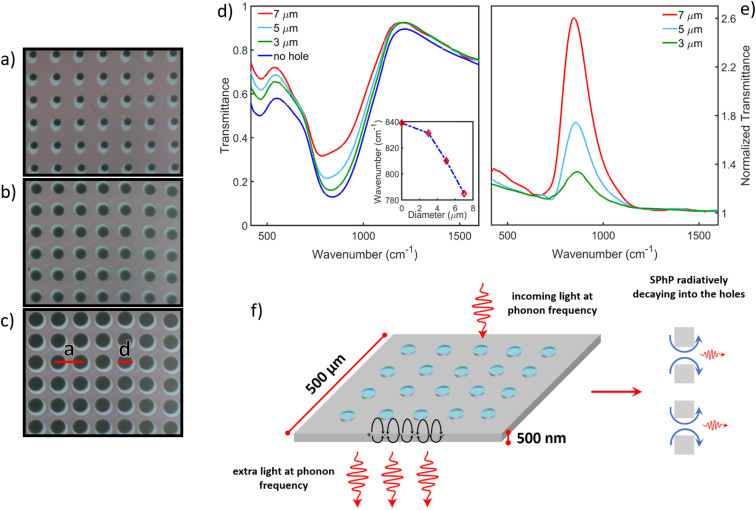
Optical microscope images of the patterned Si_3_N_4_ samples, with 3 μm (a), 5 μm (b) and 7 μm (c) hole diameters *d*, and lattice parameter *a* = 12 μm. (d) Shows the transmittance spectra for unpatterned and patterned samples. (e) Depicts instead the normalized transmittance, defined as the ratio between the transmittance of each patterned sample and the one of the unpatterned. (f) Sketch depicting the self-standing 500 nm thick Si_3_N_4_ membrane patterned with circular holes, filled with air. The membrane side is 500 μm. This picture schematically describes the EOT phenomenon, in which the incoming light at phonon frequency excites a surface phonon polariton, indicated by black arrows, trapped at the air–membrane interface. The scattering process and the radiative decay of this mode through the holes generate extra light at phonon frequency, giving rise to the EOT phenomenon, as described, for instance, in ref. 42.

In this work, we used four membranes with a thickness of 500 nm and a patterned area of 300 × 300 μm^2^ in size. The optical transmittance *T*(*ω*) of the unpatterned Si_3_N_4_ membrane has been measured in a broad spectral region from 150 cm^−1^ to 32 000 cm^−1^ to fully characterize its electrodynamic response. *T*(*ω*) is shown in Fig. 1a (light blue line). The two minima below 1000 cm^−1^ correspond to phonon modes of the membrane (see discussion below), while the numerous peaks at higher frequencies generate from a Fabry–Pérot effect related to the thin (500 nm) sample thickness. Above nearly 32 000 cm^−1^ the transmittance goes to zero due to the electronic optical gap of Si_3_N_4_. In order to extract the dielectric function *ε*(*ω*) of the material, *T*(*ω*) has been analyzed with the RefFIT software recurring to a Lorentz model,^38^ and taking into account the Fabry–Pérot effect as well. The fit is shown in Fig. 1a (black empty diamond symbols) and the experimental real (light blue line) and imaginary part (black line) of *ε*(*ω*) are represented in Fig. 1b in the same spectral range. Here, one can observe two phonon peaks at about 450 cm^−1^ and 900 cm^−1^, followed by a flat *ε*_1_ ∼ 4 region, in agreement with the absence of any electronic absorption up to the electronic gap. More specifically, the first phonon peak (at about 450 cm^−1^) is associated with the Si–N breathing mode, whereas the second peak (at about 900 cm^−1^) is caused by two (nearly degenerate) Si–N stretching modes.^39,40^ The absence of any electronic contribution in the infrared and a marked negative value of *ε*_1_ around 900 cm^−1^ strongly indicate that Si_3_N_4_ is a good material candidate for studying the phonon-polariton formation.^33,36^ Three periodic arrays of circular holes with different diameters *d* have been fabricated onto the membranes *via* Focused Ion Beam (FIB) lithography (see section 1). In Fig. 2a optical microscope images of the samples are presented, in the case of *d* = 3 μm (a), 5 μm (b), and 7 μm (c). The diameter size has been chosen to set the phononic metamaterial in sub-wavelength conditions, whereas the lattice parameter has been fixed at *a* = 12 μm to supply the wavevector required to excite the surface phonon-polariton at the air–Si_3_N_4_ interface.

Fig. 2d depicts the transmittance measurements in the phonon region for all membranes. The patterned samples show a transmittance enhancement with respect to the unpatterned one in the spectral region around 900 cm^−1^. In order to better visualize this effect, in Fig. 2e the ratios among the transmittance curves of patterned samples (green, light-blue, and red lines, in Fig. 2d) with respect to that of the unpatterned one (blue line in Fig. 2d) are presented. The extraordinary optical transmittance effect is mainly localized around 900 cm^−1^, *i.e.* in the spectral region of the Si_3_N_4_ stretching phonons. More in detail, the transmittance enhancement is on the order of 30%, 70% and 160% for the patterned samples with 3 μm, 5 μm and 7 μm hole diameter, respectively. It could be argued that the transmittance improvement is caused by holes due to a reduction in the effective refraction index of the membranes. However, this reduction would increase the transmittance across the entire spectral region, which is clearly not the case.

The EOT effect can be explained in terms of the excitation of a surface phonon polariton at the air–Si_3_N_4_ interface.^41–43^ This mode propagates along the surface and decays, when it is scattered through the holes, into a radiative channel, generating extra photons and, thus, the transmittance enhancement. A sketch summarizing the EOT effect is represented in Fig. 2f.

The inset in Fig. 2d shows the transmittance minimum frequency as a function of the hole diameter for the unpatterned (*d* = 0 μm) and patterned (*d* = 3, 5, 7 μm) samples. A redshift of these minima with respect to the unpatterned sample is observed, becoming more and more pronounced as the diameter is increased. The redshift ranges from 1% for the sample with *d* = 3 μm (nearly 10 cm^−1^), until 7% for the sample with *d* = 7 μm (nearly 60 cm^−1^). On the other hand, the transmittance minimum associated with the other phonon, around 450 cm^−1^, is not affected by a redshift at all. The redshift of the first minimum (around 900 cm^−1^) agrees with its polariton origin. Indeed, the phonon-polariton formation is related to the dielectric function sign change at the interface. While this condition is satisfied for the phonon mode around 900 cm^−1^, it is not for that at about 450 cm^−1^ (see Fig. 1b). Meanwhile, the EOT maxima depend on the diameter *d* (see Fig. 2e), their variation with the lattice parameter *a* is scarce. This result has been discussed in Fig. S1 of ESI.†

**Fig. 3:**
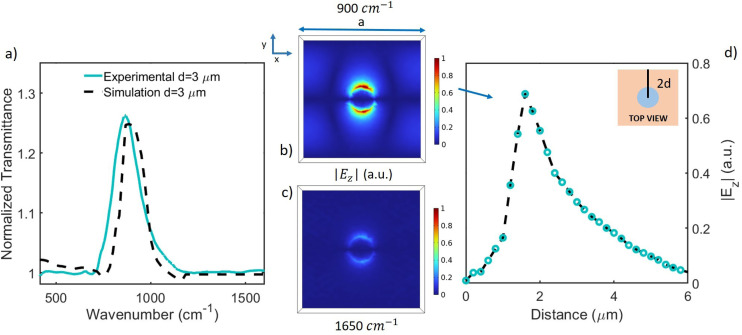
(a) Experimental (light blue line, already reported in Fig. 2e) and simulated (black dashed line) normalized transmittance for the sample with *d* = 3 μm. The simulation has been performed under an incident plane wave polarized along *y*. (b) and (c) |*E*_*z*_| distribution maps on the Si_3_N_4_ unit cell surface (*xy* plane) at a fixed height equal to the membrane thickness, in (900 cm^−1^) and out of resonance conditions (1650 cm^−1^), respectively. The electric field values were normalized between 0 and 1. (d) |*E*_*z*_| value along a line of length 2*d* (where *d* is the hole diameter), connecting the hole center (located at 0 μm) and the border of the unit cell, as shown in the corresponding inset. Light blue open circles highlight points obtained with a 200 nm spatial resolution (black dashed line is a guide for the eyes).

In order to better understand the far-field results and introduce those in the near-field (see below), numerical simulations were performed through the COMSOL Multiphysics software *via* a finite element method (see section 1). An infinite metamaterial array was simulated by drawing a single unit cell and applying periodic boundary conditions to it. Putting the dielectric function and the optical conductivity of the unpatterned membrane as input parameters, the transmittance of the bare and patterned membranes was simulated under an incident plane wave polarized along *y*. Fig. 3a shows the normalized experimental transmittance of the patterned sample with *d* = 3 μm (light blue line), compared with the simulated one (black dashed line). There is a good agreement between experiment and simulation. Similar results (not shown) were obtained for the other samples. In order to understand the electric field behavior along the membrane surface, a distribution map of |*E*_*z*_| (modulus of the electric field component along *z*) has been calculated. Fig. 3b and c show |*E*_*z*_| distribution maps on the Si_3_N_4_ unit cell surface (*xy* plane) at height *z* equal to the membrane thickness, in resonance (900 cm^−1^) and out of resonance (1650 cm^−1^) conditions, respectively. The electric field values were normalized to 1. At the resonance frequency, |*E*_*z*_| is strongly localized around the edge of the hole, while across and away from the hole it is almost zero. On the contrary, outside resonance, |*E*_*z*_| is almost zero along the whole *xy* plane. These results indicate electric field confinement around the holes is strongly enhanced in the resonant case through the SPhP excitation. Additional maps of |*E*_*x*_|, |*E*_*y*_| and 
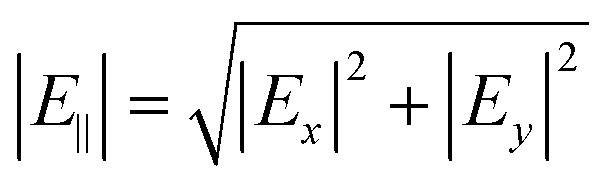
 are shown in Fig. S2 of ESI.†

Finally, Fig. 3d shows the |*E*_*z*_| value (light blue open circles), obtained at 900 cm^−1^ along a line of length 2*d* (*d* is the hole diameter), connecting the hole center and the border of the unit cell, as shown in the corresponding inset. The curve was averaged out to obtain the same spatial resolution (200 nm) of the experimental near-field measurements that will be discussed later. As expected, the field is maximized near the hole edge, decaying exponentially far from it, as expected for evanescent waves. IR nano spatially-resolved measurements were carried out at the SISSI beamline^44,45^ through the help of an s-SNOM nano-FTIR microscope (NEASPEC, Germany) coupled to an infrared tunable quantum cascade laser (QCL). The microscope allowed us to simultaneously perform IR and AFM imaging (see section 4).

**Fig. 4:**
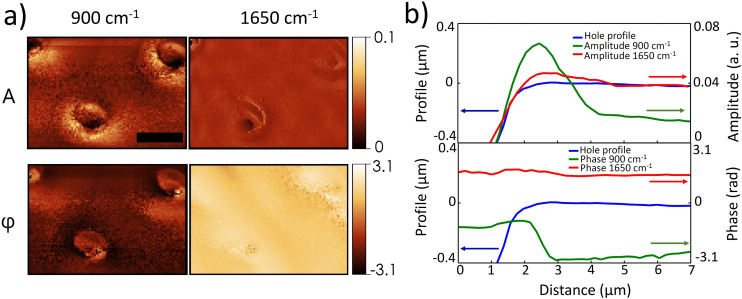
(a)Nano-IR maps of the scattering amplitude and phase variations over the *d* = 3 μm holes. On the left column, maps were obtained with infrared radiation at 900 cm^−1^, resonating with the SPhP excitation. On the right column, maps were measured with radiation at 1650 cm^−1^, out of resonance with the SPhP excitation. Maps were obtained from the same spatial region and, in order to reduce the background noise, were acquired by sampling the signal at the cantilever 3rd harmonic frequency. The black bar indicates a distance of 5 μm as a scale reference. (b) Amplitude and phase signals extracted from the maps in resonance (green line) and out of resonance (red line) of the SPhP excitation as a function of the distance from the hole center compared with the hole depth profile (blu line). The profiles shown were extracted from the hole in the upper right side.

Fig. 4 shows measurements of the scattered radiation amplitude *A* and phase *φ*, in resonance and out-of-resonance to the surface phonon excitation at about 900 cm^−1^ for the membrane patterned with holes of *d* = 3 μm. Similar data (not shown) have been obtained for the other patterned membranes. The membrane region, mapped with a spatial resolution of 200 nm, is a 12 μm × 18 μm area containing three holes of the array. The scattering amplitude and phase maps are presented in Fig. 4a, divided into two columns. On the left column, maps acquired at the SPhP excitation frequency of 900 cm^−1^ (*i.e.* in resonance to the EOT peak), are shown. On the right column, maps obtained with radiation out of resonance to the SPhP excitation (at a frequency of 1650 cm^−1^), are instead reported. Strong differences are visible between the two cases. As a matter of fact, the “out of resonance” amplitude map highlights only the morphology of the sample and the phase shows almost no variation through and away from holes. At the resonance, a strong signal variation surrounds the holes, for both amplitude and phase. These variations suggest electric field confinement due to the excitation of the SPhP, which is also present in the numerical simulations (Fig. 3b). An analogous behavior (not shown) was observed for the other two samples, with 5 μm and 7 μm holes. To better understand those maps we extracted the amplitude and phase signals as a function of the distance from the hole center, and compared them to the hole depth profile. In Fig. 4b the scattered amplitude and phase are shown, in (green line) and out of (red line) resonance conditions, together with the depth profile (blue line) as a reference. An increase in the amplitude near the edge of the hole with a strong change in the phase of ∼π/2 can clearly be seen. The amplitude curve has a very good agreement with the simulated *z*-component of the electric field shown in Fig. 3d. On the contrary, the amplitude curve out of resonance follows the morphology profile line with no change in its phase. These data confirm that the EOT phenomenon originates from the excitation of an SPhP at the air–Si_3_N_4_ interface. Noteworthy, a similar electric field confinement has been measured in nanoporous graphene, where it has been also associated with heat localization around the holes.^46^

## 4. Conclusion

In this paper, we demonstrated that the infrared electromagnetic properties of technologically relevant Si_3_N_4_ membranes can be engineered through the use of surface phonon polariton formation. Arrays of circular holes have been fabricated by the Focused Ion Beam technique on 500 nm thick Si_3_N_4_ membranes, and different hole sizes have been investigated, suggesting that the mediating mechanism responsible for the extraordinary transmittance is the excitation of a phonon-polariton mode. The electric field distribution around the holes has been further studied by numerical simulations and IR nanometric spatially-resolved measurements through a scattering-SNOM technique, confirming the phonon-polariton origin of the EOT effect. The transmittance increase in the IR spectral range paves the way for interesting sensing applications not achievable with plasmonic metamaterials due to strong optical losses in this frequency range for conventional metals.

## Author contributions

Salvatore Macis: data curation, formal analysis, investigation, methodology, software, supervision, writing – original draft preparation, writing – review, and editing. Maria Chiara Paolozzi: data curation, formal analysis, investigation, methodology, software, writing – original draft preparation, writing – review and editing. Annalisa D'Arco: investigation, supervision, writing – review and editing. Federica Piccirilli: data curation, investigation, methodology, writing – review and editing. Veronica Stopponi: investigation, methodology, writing – review and editing. Marco Rossi: funding acquisition, supervision, writing – review, and editing. Fabio Moia: methodology, sample preparation, writing – review and editing. Andrea Toma: conceptualization, funding acquisition, methodology, resources, sample preparation, supervision, writing – review, and editing. Stefano Lupi: conceptualization, data curation, formal analysis, funding acquisition, investigation, methodology, project administration, resources, supervision, writing – original draft preparation, writing – review and editing.

## Conflicts of interest

There are no conflicts to declare.

## Supplementary Material

NR-015-D3NR02834H-s001
