# Supplementary material for: Extraordinary optical transmittance generation on Si3N4 membranes
Source: Nanoscale. 2023 Sep 29;15(39):16002–9. doi: 10.1039/d3nr02834h (PMC10568977; doi:10.1039/d3nr02834h)
Supplement: NR-015-D3NR02834H-s001 [file NR-015-D3NR02834H-s001.pdf]

Electronic Supplementary Material (ESI) for Nanoscale.
This journal is © The Royal Society of Chemistry 2023

Nanoscale

Supplementary Information

Extraordinary Optical Transmittance Generation on Si_3N_4 Membranes

Salvatore Macis,*[a,b] Maria Chiara Paolozzi,[a] Annalisa D'arco,[a] Federica Piccirilli,[c] Veronica Stopponi,[d] Marco Rossi,[e] Fabio Moia,[f] Andrea Toma [f] and Stefano Lupi [a]

a Department of Physics, Sapienza University, Piazzale Aldo Moro 5, 00185, Rome, Italy. E-mail: salvatore.macis@uniroma1.it
b INFN - Laboratori Nazionali di Frascati, via Enrico Fermi 54, 00044, Frascati (Rome), Italy.
c Elettra - Sincrotrone Trieste S.C.p.A., S.S. 14 km-163,5 in Area Science Park, I-34149 Basovizza, Trieste, Italy.
d IOM-CNR, Area Science Park, Strada Statale 14, km 163,5, 34149 Basovizza TS, Italy.
e SBAI, Department of Basic and Applied Sciences for Engineering, University of Rome "La Sapienza," Via Scarpa 16, 00161 Rome, Italy.
f Istituto Italiano di Tecnologia, via Morego 30, Genova 16163, Italy.

1. EOT dependence on the lattice parameter

Extra data not shown in the main paper can help understand the behavior of the EOT phenomenon as a function of the lattice parameter *a*. By changing the hole distance *a* (for a fixed hole diameter *d*), we expect two main effects. The first effect concerns the dependence of the EOT intensity over *a*, the second one is related to the EOT central frequency dependence on *a*.

Fig. S1 describes the normalized transmittance for samples with *d*=5 µm and three different lattice parameters: *a*=12 µm (already shown in Fig. 2e of the main manuscript), and new data for *a*= 10 µm and 11 µm.

The normalized transmittance shows a clear trend: the smaller the lattice parameter, *i.e.* the hole-to-hole center distance, the larger the transmittance enhancement. Indeed, given the same patterned area (300 x 300 μm^2), the sample with *a*=10 µm shows a larger density of holes than the other two. Therefore, as roughly evident from the Figure, the total intensity of the EOT peak scales linearly with the hole density.
Moreover, as the lattice parameter a decreases, the momentum transferred by the hole array increases ($k=2\pi/a$). However, the EOT central frequency does not show a measurable shift vs. the lattice parameter, as highlighted by the black dashed vertical line in Fig. S1. A similar result (any frequency shift vs. *a*), has been observed also in a silicon dioxide hole perforated lattice (see Ref. 32 in the main text). In both cases the SPhP energy-momentum dispersion exists in a narrow spectral range, inside the Reststrahlen phonon band, and the SPhP dispersion is further reduced due the anharmonicity of the phonon mode. As changing the incidence angle of the incoming light should increase the SPhP momentum, (for the further momentum added by light), due to previous observations, no noticeable frequency shift of this excitation is expected. Finally, we can observe that our illumination angle is 30° (see Methods section in the main manuscript). This implies that we are partially sampling over the SPhP dispersion region, without observing any measurable effect.

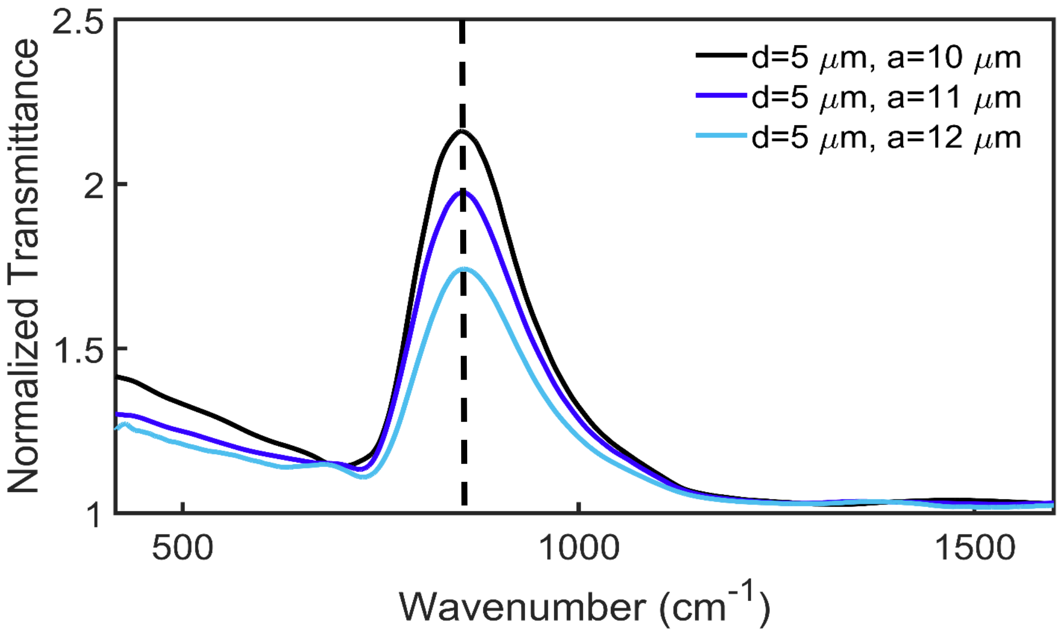

Fig. S1 Normalized transmittance, defined as the ratio between the transmittance of each patterned sample and the one of the unpatterned, for *d*=5 µm and *a*=10, 11, 12 µm.

2. In plane Electric Field maps

Together with the electric field behavior along z (Fig. 3b and 3c in the paper), we show the electric field components along the x and y directions. These maps were extracted on the Si_3N_4 unit cell surface (xy plane) at a fixed height equal to the membrane thickness, in (900 cm^{-1}) and out (1650 cm^{-1}) of resonance. Fig. S2a and S2b refer to the modulus of the electric field component along x ($|E_x|$), S2c and S2d along y ($|E_y|$), and S2e and S2f to their square modulus $\sqrt{|E_x|^2 + |E_y|^2}$. As it can be noticed, the $|E_y|$ contribution is stronger than $|E_x|$ for both frequencies, since the incoming electric field was chosen to be polarized along y. As a consequence, for the resonant case, $|E_y|$ (Fig. S2c) appears strong inside and along the hole edge, whereas, for the out of resonance case (Fig. S2d), its values are very small. The same behavior is visible in Fig. S2e and S2f since the $|E_x|$ contribution shown in Fig. S2a and b is negligible compared to $|E_y|$.

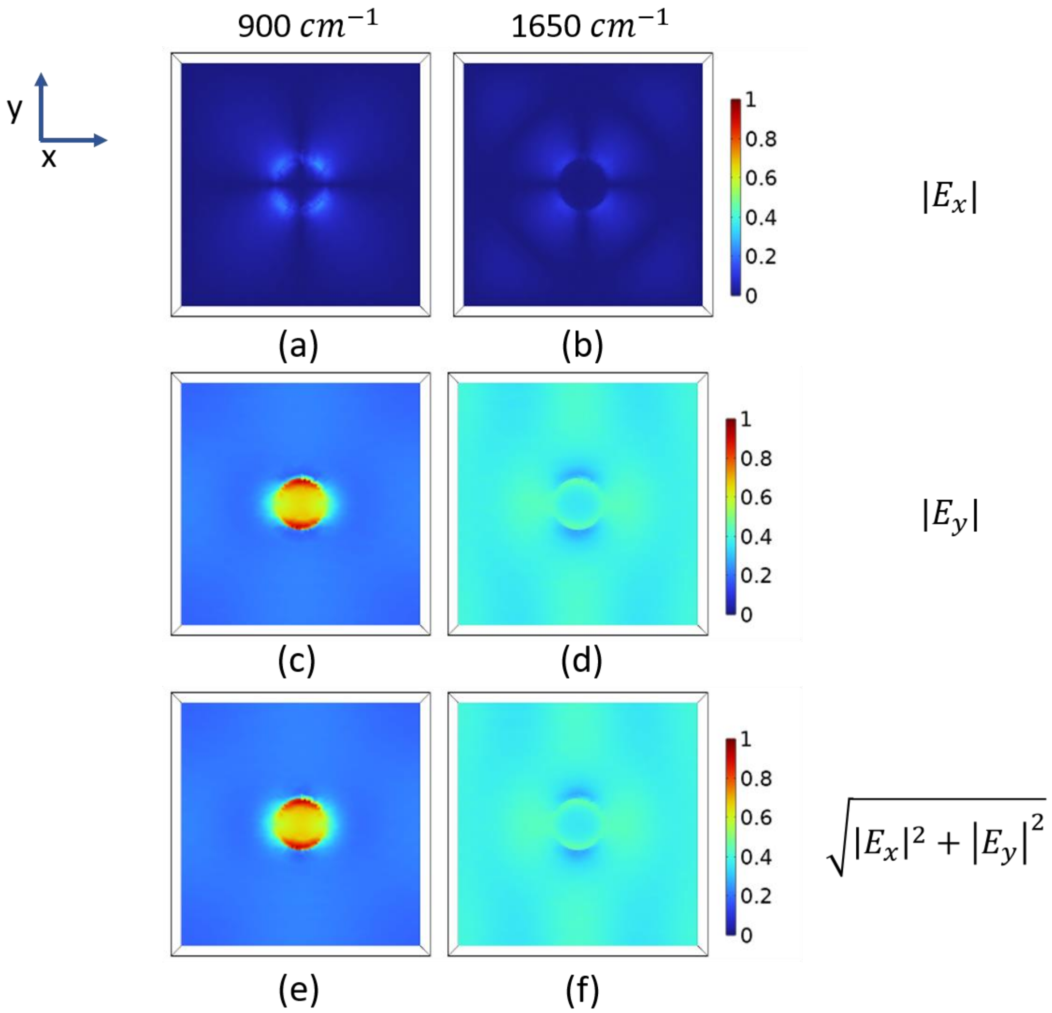

Fig. S2 Distribution maps of $|E_x|$ (a and b), $|E_y|$ (c and d) and $\sqrt{|E_x|^2 + |E_y|^2}$ (e and f) on the Si_3N_4 unit cell surface (xy plane) at a fixed height equal to the membrane thickness, in (900 cm^{-1}) and out of resonance (1650 cm^{-1}) conditions, respectively. The electric field values were normalized between 0 and 1.
